# Carotenoids in Milk and the Potential for Dairy Based Functional Foods

**DOI:** 10.3390/foods10061263

**Published:** 2021-06-02

**Authors:** Ruth Conboy Stephenson, R. Paul Ross, Catherine Stanton

**Affiliations:** 1 Vistamilk/Teagasc Food Research Centre, Moorepark, Fermoy, P61 C996 Cork, Ireland; ruth.stephenson@teagasc.ie; 2 APC Microbiome Ireland, University College Cork, T12 YT20 Cork, Ireland; p.ross@ucc.ie; 3 School of Microbiology, University College Cork, T12 YN60 Cork, Ireland

**Keywords:** bovine milk, human milk, bioavailability, bioaccessibility, infant formula, lutein, β-carotene

## Abstract

Carotenoids are a family of over 1100 known natural pigments synthesized by plants, algae, fungi and bacteria. Dietary intake of carotenoids is necessary for mammals as they cannot be synthesized in the body. In cows, the nature of the diet consumed strongly influences the composition of milk produced and this includes carotenoid concentration and profile. Fresh forage is the richest source of carotenoids for cows. The main carotenoids identified in forages are lutein, β-carotene, zeaxanthin and epilutein. Manipulating cow feed via carotenoid supplementation increases the carotenoid content of bovine milk. In humans, carotenoids have anti-oxidant, anti-inflammatory and provitamin A activity. Lutein is a major carotenoid in human milk and the brain tissue of adults and infants. Lutein and zeaxanthin are linked to improved eye health and cognitive function. Traditionally for humans, fruit and vegetables have been the main source of carotenoid intake. Functional foods present an opportunity to incorporate these naturally occurring compounds into milk products for added health benefits, widening the range of dietary sources of carotenoids. We offer an overview of the literature to date on carotenoid-fortified dairy products and infant formula. This review will describe and summarize the key mechanisms by which the carotenoid profile of bovine milk can be manipulated. We present findings on the origin and role of carotenoids in bovine and human milk, outline factors that impact the carotenoid content of milk, evaluate carotenoid-fortified milk products and discuss the associated challenges, such as bioaccessibility and stability.

## 1. Introduction

In mammals, milk is a biological fluid that provides the neonate with the required nutrients for development and immunological protection during the initial critical period of life. Bovine milk is also a popular and nutritious food item available for human consumption. Bovine milk is regularly consumed as part of a balanced diet and is an important source of various macronutrients, including protein (~3.2%), lactose (~4.8%) and fat (~3.5%) [1]. Furthermore, bovine milk also provides micronutrients such as vitamins (A, D, B, C), minerals (calcium, magnesium, potassium) and trace elements (zinc, iodine, copper) [2]. Milk is also a unique source of bioactive compounds, including carotenoids. Carotenoids can be found within the lipid fraction of milk [3] and contribute the characteristic yellow hue associated with dairy products [4].

Carotenoids are naturally occurring lipid-soluble plant pigments that are only obtained by mammals through their diet. Carotenoids have valuable anti-oxidant and anti-inflammatory properties [5]. Carotenoid consumption has been linked with various health-promoting effects including improved ocular health and cognitive function [6,7] and provitamin A activity [8]. In the human diet, carotenoid intake is mainly associated with fruit and vegetable consumption. In recent years, there has been increased interest in alternative dietary sources of carotenoids through the development of functional foods. While there is no globally accepted definition for functional foods for the purpose of this review, functional foods are whole, fortified, enriched or enhanced foods that confer positive health effects to the human body [9]. Furthermore, functional foods should take the form of ordinary foods and not that of a pill or capsule [10]. The existing carotenoid content of bovine milk, in combination with its valuable composition of macro- and micronutrients, highlights the potential for a fortified dairy product. Three servings of dairy a day are recommended by the Irish Department of Health as part of a balanced healthy diet [11]. The major barriers to carotenoid fortification include low stability, bioaccessibility and bioavailability and potential sensory changes to the final product [12]. The unique fat composition of dairy may increase the stability and bioavailability of carotenoids. Dairy fortification presents an opportunity for the food and farming industry to target milk for increased nutritional value. When developing a functional food product, it is important to consider consumer opinions, with current preferences for a naturally sourced and nutritionally valuable product [13]. This complements the naturally occurring nature of carotenoids and high nutritional value of milk. Carotenoids are also important bioactive constituents in human milk and have been implicated in infant health and development [14]. It is important after birth that the infant receives the correct nutritional supply to ensure proper development and immunological protection. However, to date, little emphasis has been placed on carotenoid inclusion in infant formula preparations [15].

This review will discuss the origins of carotenoids in bovine milk and the potential to modify the carotenoid profile of milk for a dairy functional food. The carotenoid content of milk can be manipulated in three ways—firstly, on the farm via choice of feeding system and farm management; secondly, on the farm via supplementation of the dietary feed; and finally, by supplementation of the milk or milk product at a later stage in the manufacturing process. We will present the main findings on carotenoid fortification of milk with reference to the current challenges faced both at the farming level and during product development. We will also examine the potential to fortify infant formula to ensure adequate carotenoid supply for the infant when mothers’ own milk may not be available.

## 2. Carotenoids

Carotenoids are a family of over 1100 naturally occurring pigments found in plants, algae, fungi and bacteria [16]. Carotenoids are divided into two classes based on their chemical composition—xanthophylls and carotenes. Carotenes are exclusively composed of hydrogen and carbons, whereas xanthophylls also contain oxygen. Both classes are lipophilic.

In plants, carotenoids have a functional role in photosynthetic processes and confer a yellow-red color to tissue, acting as attractants in various fruit and flowers [17]. In mammals, carotenoids are associated with various health-promoting effects, mainly as anti-oxidant [5,18,19], anti-inflammatory [18,20] and immunomodulatory [21,22] compounds. Carotenoids with an unsubstituted β-ionone ring have provitamin A activity, mainly carotenes (β-carotene, α-carotene) but also the xanthophyll β-cryptoxanthin. Vitamin A contributes to vision and growth and is one of the major nutrient deficiencies worldwide, particularly in developing countries [23]. Xanthophylls, particularly lutein and zeaxanthin, have been associated with improved ocular and cognitive health in humans. Lutein and zeaxanthin preferentially accumulate in the macula of the retina with meso-zeaxanthin, i.e., lutein’s metabolite, to compose the macular pigment [6]. The macular pigment acts as an optical filter for blue light (400–500 nm), protecting the retina from photochemical damage [24]. Increased lutein and zeaxanthin intake are associated with reduced risk of age-related macular degeneration, the leading cause of blindness in the Western world [25]. In the adult brain, xanthophylls account for 66–77% of total carotenoids [26,27]. In older adults, these unique bioactive compounds have been linked to improved cognitive health [7,27,28]. Furthermore, reduced macular pigment, lutein and zeaxanthin serum levels were reported in patients with Alzheimer’s disease [29].

In mammals, carotenoids cannot be synthesized de novo and are only obtained through diet. For this reason, and taking into account that carotenoids have many biological functions in humans, an adequate concentration in dietary sources is important. In total, 40–50 carotenoids have been identified in common foodstuffs, primarily fruit and vegetables [30]. The major carotenoids in food include lutein, β-carotene, α-carotene, zeaxanthin, β-cryptoxanthin and lycopene [30,31]. Furthermore, violaxanthin, neoxanthin, phytoene and phytofluene have also been detected in a variety of dietary sources [32]. Green vegetables are known for their particularly high lutein concentration, with concentrations of 44–395 and 48.10–119.38 µg/g for kale and spinach, respectively [33,34]. Carotene levels are high in yellow/red fruits and vegetables [30]. Beta-carotene concentrations in pumpkin and carrots are 17–422.6 and 34–182.5 µg/g, respectively [31,34]. Similarly, in carrots, α-carotene levels of 22–106.5 µg/g have been recorded. Lycopene is found in high levels in red fruit, like tomatoes (25–233 µg/g), in addition to phytoene and phytofluene [31,35,36]. Similar to fruit and vegetables, egg yolk has high lutein and zeaxanthin concentrations, with an average total xanthophyll content of 12 µg/g [33]. Ollilainen et al. reported even higher levels of lutein, 15.76 µg/g [37]. Bovine milk is a known source of carotenoids, particularly β-carotene. The concentration of carotenoids in dairy products from Finland was quantified [37]. The β-carotene contents in milk and full fat cream were 16.7 and 186.5 µg/100 g, respectively. In cheese, β-carotene content varied with type, ranging from 13.7 to 186.5 µg/100 g. Lutein was found in trace amounts in all products and the fat content affected β-carotene concentration. As carotenoid intake is only achieved through the diet, increasing the supply of carotenoids via food fortification is an area of interest, particularly due to their potential health attributes.

## 3. Functional Foods

**Figure 1:**
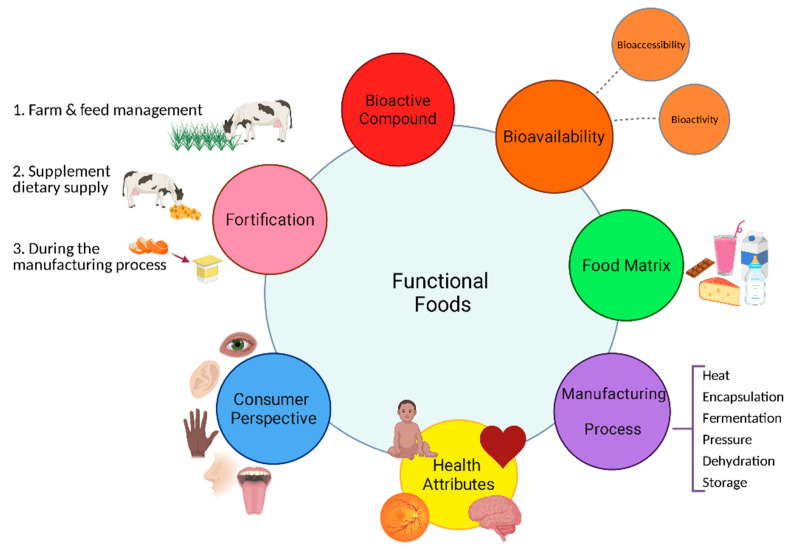
Examples of the key concepts to consider when designing a carotenoid functional food, in particular a dairy product with added value. Each factor can potentially influence another and all these interactions must be considered. This figure was created using BioRender.com (accessed on 24 March 2021).

When designing a carotenoid-fortified food, a variety of factors must be considered to ensure optimal carotenoid delivery and a marketable food product with enhanced value for the consumer (Figure 1). The low bioavailability, solubility and stability of carotenoids are key challenges facing the food industry when developing a fortified food product. Bioavailability refers to the “fraction of the nutrient or bioactive compound ingested that is available for use in physiologic functions or to be stored” [38]. The bioavailability of functional foods is generally determined by measuring the carotenoid levels in plasma. Bioavailability is distinguishable from bioaccessibility which is “the fraction of a compound that is released from its matrix in the gastrointestinal (GI) tract and thus becomes available for intestinal absorption” [38]. The bioaccessibility directly impacts the bioavailability and the subsequent bioactivity of carotenoid-fortified products in vivo. In order to design a functional food, an understanding of the challenges and contributors to low carotenoid bioavailability is vital. A detailed review on the absorption of carotenoids and factors limiting bioavailability including, food matrices, carotenoid–nutrient interaction and processing steps has recently been published [12]. This section will give an overview of carotenoid digestion, absorption and metabolism in vivo. It will also look at the importance of the food matrix in the context of dairy, focusing on fat content. The fat content in dairy could be utilized to overcome the challenge of low bioavailability associated with carotenoids in fruit and vegetables. Bovine milk and dairy are commonly consumed and a popular food group with potential for high carotenoid delivery.

### 3.1. Digestion, Absorption and Metabolism of Carotenoids

For carotenoids to carry out their biological functions in vivo, these bioactive compounds must successfully reach their target tissue following ingestion. The first step is the release of carotenoids from the food matrix by mastication and enzymatic action in the mouth before undergoing emulsification into lipid droplets in the stomach [39]. Carotenoids are then transferred into mixed micelles and transported to the apical surface of epithelial cells lining the GI tract, ready for intestinal absorption [12]. The portion of carotenoids incorporated into mixed micelles and ready for uptake by intestinal absorptive cells is known as ‘bioaccessibility’ [40]. The ‘bioavailability’ of carotenoids is the level of successfully absorbed carotenoids in the plasma ready for utilization by the body.

Previously, intestinal absorption of carotenoids was thought to occur via passive diffusion. However, in vitro studies suggest the role of various lipid transporters (Table 1) [41]. To date, three lipid transport proteins have been implicated in this process, Scavenger receptor class B type I (SR-BI), Cluster Determinant 36 (CD-36) and Niemann–Pick C1-Like 1 (NPC1L1) [42].

Following transfer to the cell interior, provitamin A carotenoids undergo transformation to retinol or retinyl esters which are packaged into chylomicrons, in addition to non-provitamin A carotenoids [12]. The mechanism of intracellular uptake of carotenoids by chylomicrons remains unclear [51]. Chylomicrons are secreted into lymph before release into plasma. In the plasma, chylomicrons are reduced by lipoprotein lipase to chylomicron remnants and undergo uptake by hepatocytes. In the liver, carotenoids are incorporated into very-low-density lipoproteins (VLDL) particles and secreted back into the plasma, where they are circulated as low-density lipoproteins (LDL) for peripheral distribution to their target tissue via LDL receptors [39].

The steps involved in bioaccessibility and the factors capable of influencing carotenoid bioavailability should be considered when designing a fortified food product to ensure the bioactive potential of carotenoids is fully realized. These factors are represented by the mnemonic SLAMENGHI (S: species of carotenoid; L: molecular linkage; A: amount consumed in a meal; M: matrix used for carotenoid incorporation; E: effectors of absorption and bioconversion; N: nutrient status of the host; G: genetic factors; H: host-related factors; I: interactions) [52]. In particular, this review will examine the choice and quantity of carotenoid species, and their interaction within a dairy based matrix in studies to date.

### 3.2. Dietary Factors Impacting Bioavailability

The dietary components of food can significantly affect the absorption and availability of carotenoids. Dietary lipids enhance carotenoid absorption from fruit and vegetables which are naturally low in fat [53,54]. Dairy products are a potential vehicle for increased carotenoid delivery due to the fat content of milk which is composed of 65% saturated fatty acid (SFA), 32% monounsaturated fatty acid (MUFA) and 3% polyunsaturated fatty acid (PUFA) [55].

The co-ingestion of dietary fat facilitates the solubilization of carotenoids following release from the food matrix and enhances carotenoid micellarization [56]. Lipid intake prompts the secretion of bile salt and pancreatic enzymes, which contribute to lipid digestion and micelle formation [41]. Additionally, lipid digestion provides increased digestive products for the structuring of mixed micelles, including lysophospholipids, monoglycerides, and free fatty acids [50]. Overall, dietary lipid intake improves micelle formation and carotenoid uptake, ultimately increasing the bioaccessibility of the micronutrient. A study by Mashurabad et al. [56] examined the effect of vegetable oil intake on carotenoid (lycopene, α-carotene, β-carotene and lutein) micellarization in various fruit and vegetable food matrices (carrot, spinach, drumstick leaves and papaya). They reported that dietary fat intake of 1–2.5% is sufficient to enhance carotenoid micellarization and saturation occurs at 5% [56]. Additionally, dietary fat provides increased products of lipid digestion for chylomicron assembly [51], ultimately enhancing carotenoid transfer to the lymphatic system.

Lipid type with regard to fatty acid chain length and degree of unsaturation is an influencing factor in carotenoid absorption. The micellarization of carotenoids from some vegetables and fruits was 2–3 times higher in fat sources with higher levels of unsaturated fatty acids compared to SFAs [56]. Clark et al. reported higher carotenoid absorption in olive oil (MUFAs) than corn oil (PUFAs) [57]. Furthermore, Borel et al. observed decreased β-carotene and astaxanthin uptake by chylomicrons in the presence of medium-chain triglycerides in comparison with long-chain triglycerides, suggesting decreased chylomicron secretion in the presence of medium-chain triglycerides [58]. In a study with spinach, bioaccessibility of lutein was highest when combined with fat sources rich in medium-chain SFAs and highest for β-carotene in the presence of long-chain MUFAs [59]. These findings highlight the influence of fat type and a specific interaction of carotenoid class on bioaccessibility. Furthermore, the amount of dietary fat intake, the food matrix and the polarity of carotenoids impact carotenoid micellarization and subsequent absorption [56].

Evidently, dietary fat intake, fat type, food matrix and choice of carotenoid should be considered when designing a fortified product for optimized bioactivity in vivo. Specifically for dairy product fortification, the interaction of the class and polarity of carotenoids with the type and amount of fat should be assessed. The unique lipid content of a dairy food-based matrix may alleviate the limiting factors associated with low bioavailability of carotenoids in foodstuffs.

## 4. Bovine Milk

### 4.1. Carotenoids in Bovine Milk

**Figure 2:**
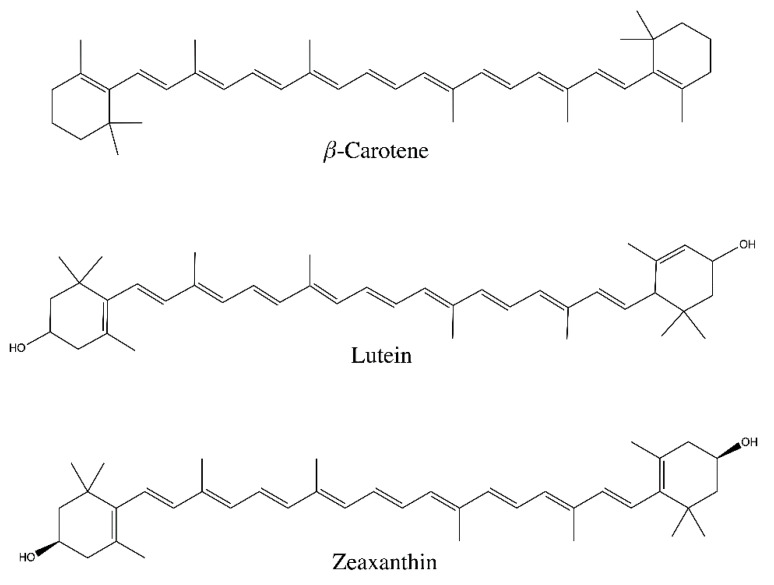
The main carotenoids identified in bovine milk.

Carotenoids are stored in the lipid fraction of bovine milk. Only a small number of carotenoids have been identified in bovine milk, with β-carotene and lutein representing the two major carotenoids (Figure 2) [60]. Of these, β-carotene is the dominant carotenoid, comprising 75–90% of the total carotenoid concentration [60,61,62]. Zeaxanthin was identified at low levels in milk [63]. However, it was not identified in a separate study [60].

### 4.2. The Health-Promoting and Sensorial Effects of Carotenoids in Bovine Milk

Beta-carotene, a major milk carotenoid, is a vitamin A precursor [8]. The high concentration of β-carotene enhances milk’s natural properties and provides a valuable source of vitamin A to the consumer. In bovine health, carotenoid consumption has been linked with reduced incidence of post-calving reproductive disorders [64] and improved udder health and fertility [65,66].

Carotenoids also influence the sensorial properties of bovine milk in two ways. Firstly, they confer the yellow hue associated with dairy products. The yellow color is more strongly associated with grass-based feeding systems [4]. The yellow color of dairy based products is important in terms of consumer preference, traditionally indicating naturally farmed milk. The potential for the yellow color to act as a biomarker for feed management and as an indicator for carotenoid (mainly β-carotene) concentration has been suggested [67], although other findings suggest that the color index of bovine plasma, not milk, may be a better indicator [60]. Secondly, the anti-oxidant capacities associated with carotenoids have protective effects against oxidation of milk [68]. Carotenoids act as scavengers of singlet oxygen and their ability to act as light filters has previously been demonstrated [69,70]. These findings suggest the potential for β-carotene to protect milk from light-activated oxidation as a result of riboflavin sensibilization. However, Havemose et al. found that increased carotenoid levels did not prevent the sensibilization of riboflavin or reduce lipid oxidation. Instead, higher carotenoid content was associated with lower protein oxidation, which results in the initial off-flavors attributed to light-induced oxidation [71]. Evidently, carotenoids in bovine milk influence the nutritional quality and stability of milk due to their provitamin A action, anti-oxidative capabilities and potentially immune-enhancing effects.

### 4.3. Factors Influencing the Carotenoid Content of Bovine Milk

As outlined in Section 4.2, milk is a valuable source of carotenoids that contribute to its nutritional and sensorial properties. Bovine milk relies on external dietary sources for carotenoids and the choice of forage influences dietary supply. This section will give an overview of the influencing factors and potential mechanisms which could alter the carotenoid levels of milk and subsequent dairy products at the farm level. A variety of physiological factors are known to impact milk composition including genetics, the health status of the cow, the stage of lactation and parity [1]. These factors have also been associated with influencing carotenoid uptake and concentration in milk [72]. In particular, an effect of breed has been reported [63,73,74].

#### 4.3.1. Dietary Sources for Bovine Carotenoid Consumption

Bovine carotenoid consumption is entirely dependent on dietary supply, which is mainly forage-based. To date, less than 10 carotenoids associated with forage feed have been identified [72]. These include the β-carotene isomers, 13-*cis* and all-*trans*, in addition to 5 of the xanthophyll class, violaxanthin, antheraxanthin, lutein, zeaxanthin and epilutein, each representing 4%, 11%, 14%, 3%, 49%, 10% and 9% of total carotenoid content, respectively [61]. Carotenoid levels vary with herbage types. Investigations by Larsen et al. reported higher β-carotene and lutein levels in a red clover mixture (235 and 655 mg/kg of dry matter (DM), respectively) in comparison with a lucerne mixture (148 and 433 mg/kg of DM, respectively) [75]. Although Cardinault et al. reported lower levels of lutein and β-carotene in red clover (136 and 29 of µg/g of DM, respectively) [76], finding by Livingstone et al. support a higher concentrations of xanthophylls and carotenes in red clover in comparison with lucerne [77]. Elgersma and colleagues also reported disparity in carotenoid content between forage types ranging from 129 to 206 mg/kg of DM for lutein and 26 to 61 mg/kg of DM for β-carotene [78]. Carotenoid content is lower in preserved forage and relative to the portion of grass-based forage [79,80]. The ensiling process reduced β-carotene in Virginia Fanpetals by 56% [81]. Grass-based silage has higher total carotenoid concentrations than hay, 441 and 165 µg/g of DM, respectively [60]. In the same study, there was a 50% reduction in lutein from hay vs. grass silage [60]. Other factors that influence the carotenoid content of forage are fertilization [82,83,84], the ratio of leaf to stem [77], diurnal variations [17], cutting height [81], harvest date and maturity [83,85,86].

Clearly, forage choice and feed management can strongly influence the level of carotenoid ingestion and therefore, carotenoid output in bovine milk. An extensive review on carotenoids in forage and their transfer to bovine tissue and milk has previously been carried out [72].

#### 4.3.2. Feed Management

Production conditions influence the nutritional profile of milk. In particular, the effect of different feeding systems on carotenoid concentration has been established. The two main feeding systems utilized by the farming industry are pasture-based feeding (grazing fresh grass and forage) or total mixed ration (TMR) feeding. For a TMR-based feeding system, cows are usually housed indoors and supplied with grass/maize silage supplemented with high levels of concentrate feeds [87]. The feeding system employed by farmers is determined by a number of factors including climate, environmental and economic considerations. A pasture-based feeding system is widely used by the dairy industry in temperate regions like Ireland and New Zealand [88]. Pasture-based feeding has positive effects on the nutritional composition of bovine milk [3]. Additionally, it is a cheaper source of feed than more expensive concentrate feeds. Dillon et al. reported that milk production costs are reduced by 2.5 cent per liter for every 10% increase in the proportion of grazed grass [89]. It is important to note that in the majority of studies to date, the effect of season is often interchangeable with dietary effects. This is a result of indoor housing and preserved feeding during the winter period versus cows grazing outdoors on pasture in the spring and summer months.

As outlined in Section 4.3.1, fresh pasture is the optimal source of carotenoids for ruminants. Feeding fresh herbage increases the carotenoid content of bovine milk, in comparison with conserved forage and concentrate feeds. A study of bulk tank raw milk in the Netherlands demonstrated that winter feeding resulted in a 20% reduction in β-carotene [62]. Furthermore, the total carotenoid levels of raw milk were 16.8 µg/100 g following the winter period as opposed to higher levels of 24.5 µg/100 g recorded following the summer months. This decrease was attributed to cows being fed a mixture of grass and corn ensilage and pellets. Similarly, French bulk tank milk has higher β-carotene and lutein during the summer months with increases of 2.0 and 0.23 µg/g of fat, respectively, in addition to a stronger yellow color [4]. The highest levels of β-carotene were observed in September (5.3 µg/g fat) and for lutein in July (0.59 µg/g fat). Furthermore, a UK-based study recorded increased lutein and zeaxanthin concentrations in milk from grazing systems with greater proportions of pasture-based feeding over a 2 year period [63]. Preserved forage feeding decreases the carotenoid content of bovine milk. However, during the winter period, indoor feeding of preserved forage and concentrates is necessary due to poor weather. The proportion of grass-based preserved forage and method of forage conservation impact the carotenoid profile of milk. Calderon et al. demonstrated that a switch in diet from hay to grass ensilage and alfalfa protein feed resulted in increased β-carotene from 2.74 to 4.21 µg/g of fat [60]. The lutein levels remained constant despite dietary changes averaging 0.73 µg/g of fat. Similarly, a shift from grass ensilage to hay resulted in a rapid decrease in β-carotene levels from 0.17 to 0.07 µg/mL [74]. Furthermore, adjusting the ratio of maize to lucerne silage from 5:1 to 2:1 resulted in a 23–27% increase in carotenoid concentration of milk fat [90].

It is apparent that farm management, particularly feeding systems, is an accessible and effective way to modify the quality of milk and dairy products. Thus, the farming and dairy industry can potentially target milk production for the development of milk and dairy products with different nutritional profiles, including carotenoids.

#### 4.3.3. Grassland Management

Forage maturity influences the carotenoid content of milk. Calderon et al. noted a significant decrease in the carotenoid concentration of milk (5.37 to 3.87 µg/g of fat) during the growth period, which was followed by an increase in early regrowth to 4.91 µg/g [61]. These findings indicate that herbage maturity does affect the carotenoid concentration of milk. This is likely a result of the decreased carotenoid content of maturing forage, as noted in Section 4.1. Additionally, the same group examined the effect of different grazing patterns, rotational vs. strip grazing. Grazing management did not significantly impact the β-carotene concentration in milk, whilst minor reductions in lutein levels were observed following rotational grazing [61]. Although it is not a major factor, forage maturity can be considered when supplying feed to bovines for optimal carotenoid output in milk.

#### 4.3.4. Microbial Rumen Degradation

The effects of rumen digestion on the bioavailability of carotenoids remain unclear. During rumen digestion, carotenoids are released from the plant matrix and incorporated into the rumen liquid phase. This is followed by the absorption and transportation of carotenoids to peripheral tissues for metabolism, which occurs in a tissue-specific manner (adipose storage, provitamin A and anti-oxidant activity) [72]. The participation of rumen microbes in carotenoid degradation remains uncertain due to varying results from in vivo and in vitro studies, ranging from high levels of degradation to low [72]. In bovines, rumen degradation of carotenoids has been shown to occur at low levels and losses were not associated with attack by rumen micro-organisms [91,92]. Furthermore, studies have suggested a positive interaction between carotenoids and rumen microbes. A study in sheep demonstrated that carotenoid levels (lutein and β-carotene) increased following ruminant digestion [76]. Carotenoid intake was 174, 16.5 and 20.9 mg/day for lutein, 13-*cis*-β-carotene and *trans*-β-carotene, respectively. By the stage of duodenum analysis, levels had increased to 204, 53.4 and 63.9 mg/day, respectively. The authors proposed that increased carotenoid levels occurred as a result of rumen microbes facilitating the release and synthesis of carotenoids in the rumen. As noted in their study, previous findings that micro-organisms have the ability to synthesize carotenes and the presence of β-carotene in fecal samples from calves deprived of carotene, following inoculation with rumen microbes, supports their theory [76]. Additionally, Hino et al. demonstrated the ability of β-carotene to increase bacterial growth following suppression by safflower oil in the ruminal content of goats [93]. Similarly, β-carotene enhanced microbial growth in goat rumen fluid [94]. The potential for a mutualistic relationship between the microbial population in the rumen and carotenoid ingestion exists. Evidently, this relationship warrants further exploration to fully understand the digestion of carotenoids and the potential to increase carotenoid concentration in vivo. Future studies should also examine whether carotenoids influence the transformation of other digestive products. Overall, these findings suggest a positive role of rumen microbes in carotenoid digestion.

#### 4.3.5. Saturation Phenomenon in the Carotenoids Transfer from Plasma to Milk

The final concentration of carotenoids in bovine milk relies on the transfer from plasma to milk via the mammary gland. This occurs at relatively low levels. Noziere et al. demonstrated low daily extraction rates of β-carotene of 0.008% [74]. Plasma concentration of carotenoids varies but is higher than that of milk. Plasma levels of β-carotene were 5.10 µg/mL and 1.71 µg /mL following grass silage and hay feeding, respectively. In comparison, the levels in milk were 30 and 24 times lower, respectively [74]. Low levels of β-carotene transfer occur even in the presence of a carotenoid-rich diet. Investigations by Calderon et al. noted that saturation is a contributing factor influencing β-carotene uptake under high carotenoid dietary conditions. In this study, high levels of β-carotene feeding resulted in a plateau in bovine milk. The β-carotene concentration of milk fat following 28 days of carotenoid-rich feed did not differ between groups despite varying rations. This trend was not observed in bovine plasma concentrations. The group suggest that β-carotene levels of 5 µg/mL in plasma result in saturation and further increments in β-carotene supply will not increase β-carotene yield in milk [60]. Findings by Agabriel et al. [4] and Larsen et al. [75] support this theory. Agabriel and colleagues, 2007 [4] noted that a shift from a period with low carotenoid intake (February-March) to higher carotenoid intake did result in a significant increase in the milk β-carotene concentrations. However, within the period of higher carotenoid intake (May-September), an increase in the proportion of β-carotene content did not result in any significant changes [4]. Findings suggest that saturation is a limiting step in the uptake of β-carotene in the mammary gland and subsequent secretion into the milk. The exact mechanisms of carotenoid uptake in the mammary gland needs to be explored further. This phenomenon should be considered when manipulating carotenoid concentration, particularly β-carotene, at the farm level.

## 5. Dairy as a Functional Food

Carotenoids are desirable components in bovine milk and subsequent dairy products. The potential for a dairy functional food as a vector for increased carotenoid consumption is a growing area of interest. The ability to manipulate the carotenoid content and profile of dairy via farm management or supplementation both at the farming and processing stages has been established. Of particular interest are the carotenes with provitamin A activity and lutein and zeaxanthin due to their associated health benefits. When developing a carotenoid-fortified food current safety recommendation should be considered. The European Food Safety Authority (EFSA) has advised an acceptable daily intake (ADI) of 1 and 0.5 mg/kg of body weight for lutein and lycopene, respectively [95,96]. Although there are no current ADIs for β-carotene and zeaxanthin, and EFSA report no safety concerns when daily supplemental intake levels remain under 15 and 0.75 mg/kg of body weight, respectively [97,98]. Furthermore, various carotenoid containing products have been accepted as GRAS (generally recognized as safe) by the US Food and Drug Administration (FDA), these findings have previously been reviewed [99]. The advised levels are higher than the average daily consumption of carotenoid (5.42–15.44 mg/day) and indicate the potential for carotenoid fortification of food products with safe and beneficial outcomes [22].

The modification of the carotenoid profile of milk and dairy products via farm feeding practices has been reported. Butter produced from pasture-based milk had higher levels of *trans*-β-carotene than butter derived from TMR-based milk, 5.16 and 2.27 mg/kg, respectively [100]. Additionally, the pasture-based butter had enhanced sensorial properties (flavor and appearance). This highlights the potential to naturally fortify milk through farm management without negatively impacting the sensorial attributes of the dairy product. This is supported by the previously discussed findings that dietary supplementation and farm management practices can influence the carotenoid content of milk [4,62]. Lutein supplementation of cow feed increased the lutein content of milk [101,102]. Xu et al. reported that the incorporation of lutein into TMR at 0–4 g/day per head resulted in the lutein concentration of milk increasing from 0.59 to 1.50 µg/100 mL. Lutein supplementation also improved milk yield and quality [101]. Furthermore, supplementation of bovine feed with lutein and lutein co-supplemented with vitamin E increased the lutein levels of milk by 3.8 µg/L and 5.66 µg/L, respectively [102]. In this case, lutein supplementation had positive sensory effects on texture, flavor and aroma with slightly lower scores for appearance. Dietary supplementation on farm is a viable option and warrants further exploration to elevate milk carotenoid content and to determine sensory attributes, optimal concentration and stability.

Dairy products can be considered for functional food development. However, in order to design a product that optimizes carotenoid bioactivity, the barriers in terms of bioaccessibility, bioavailability, safety, stability and sensorial properties need to be overcome. These challenges in relation to the fortification of dairy products to date will be discussed in this section. Although it is not within the scope of this review, plant-based milk alternatives originating from soy, oat, coconut, hemp, rice and nuts have grown in popularity in recent years [103]. Despite attempts to mimic the nutritional and sensorial properties of bovine milk, plant-based alternatives often have poorer nutritional value and are highly variable [104,105]. For instance, the average protein content in 17 plant-based alternatives was only 48% that of bovine milk [104]. Additionally, in an Australian-based cross-sectional survey, only 1/3 of plant-based alternatives had similar calcium concentrations to bovine milk [106]. The carotenoid content and profile of plant-based milk has largely been overlooked in studies to date despite its nutritional benefits and unique sensorial contribution to bovine milk. Future analysis and development of plant-based milk should examine the inclusion of carotenoids and their behavior in a plant-based matrix.

### 5.1. Bioaccessibility

Establishing the potential bioaccessibility of a bioactive compound in a food product should be of priority when designing a fortified food. The nature of the lipid in dairy makes it an ideal candidate for increasing carotenoid transfer to micelles and subsequent absorption in vivo. To date, information on carotenoid bioaccessibility in dairy based produce is limited but promising.

Xavier and colleagues investigated the in vitro bioaccessibility of lutein esters in milk and yogurt [107]. Lutein esters were added to whole, semi-skimmed and skimmed milk, in addition to their yogurt counterparts. The study reported similar findings for both milk and yogurt products. The bioaccessibility of lutein was 38.3–47.5% in whole and semi-skimmed dairy products, with significantly lower accessibility in skimmed products (17.8%). Furthermore, low levels of carotenoid micellarization were observed in skimmed milk (19.7%) in comparison with whole and semi-skimmed, 46.5% and 45.8%, respectively. The authors proposed a minimal fat requirement of 1.55% for carotenoid micellarization and absorption. Similarly, when persimmon fruit was added to yogurt as a source of carotenoids, bioaccessibility improved by 21% in the presence of a higher fat content of 3.6% vs. 0.25%. In the presence of whole milk, provitamin A carotenoids had a bioaccessibility of 38% [108]. This highlights the potential to combine carotenoid-rich fruit with dairy products for a fortified food product. Furthermore, improved bioaccessibility of total carotenoids and β-carotene, β-cryptoxanthin, zeaxanthin and lutein was reported in a whole milk-fruit juice combination, with reductions of 72–17% in the skimmed milk alternatives [109]. In a similar study, a fruit juice-whole milk combination had higher bioaccessibility, 23.5%, than the soy- or water-based substitutes, 15.9% and 12.9%, respectively [110]. Again, this is likely due to the higher fat content in a milk-based food matrix (3.6%), in comparison with soy (1.6%) and water (0%).

Overall, studies to date demonstrate good bioaccessibility of carotenoids in a dairy based food matrix, ranging from 23.5 to 47.5%. Findings suggest that the lipid portion of dairy is a key factor contributing to improved carotenoid bioaccessibility. Based on the findings above, a fat content of approximately 1.55% is sufficient to ensure optimal carotenoid bioaccessibility. When targeting consumers conscious of the natural origins of food products, the fortification of dairy with the naturally occurring carotenoids in fruit and vegetables is ideal. Similarly, increasing the carotenoid content through farming practices may also be viewed favorably. Future studies should examine the bioaccessibility of fortified dairy products following modification at the farm level.

### 5.2. Stability

The stability of carotenoids in dairy is a concern when designing and marketing a fortified food product. It is important that the levels of carotenoid remain constant during storage prior to consumption and that the sensorial properties are unaffected. In a study with strawberry yogurt it was indicated that up to 3 mg of lutein can be incorporated in to a 170 g portion of yogurt without any significant change in the sensory characteristics of the fortified product [111]. Furthermore, the physicochemical properties including pH, viscosity, flavor and texture remained unchanged. A slight decrease in lutein was observed over a 5 week storage period at 5 °C [111,112]. In contrast, Domingo and colleagues demonstrated that lutein added to yogurt remained stable during storage for 35 days at refrigeration temperatures [113]. Furthermore, lutein also protected the yogurt product from photo-oxidation during storage and scored well during sensory evaluation. Findings indicate that lutein is stable as part of a fortified yogurt product. The incorporation of lutein into cheese has also been evaluated. Lutein was added to Cheddar cheese at 1, 3 and 6 mg per serving size (28 g). No lutein degradation was observed over 24 weeks of ripening at 4.5 °C, confirming the stability of lutein during the aging process [114]. Although no differences in body/texture and sensory/color were noted during sensory evaluation, lutein fortification did result in a distinct bitter flavor and changes in appearance [114]. Similarly, the successful fortification of Prato cheese with lutein has been demonstrated with varying sensorial effects [112,115]. Lutein remained stable during the cheese-making process, with high levels of lutein recovery (95.2%) [112]. Kubo et al. reported an intense yellow-orange color and bitter flavor following fortification. In contrast, Sobral and colleagues noted that lutein supplementation of Prato cheese without annatto remained stable during ripening for 60 days at 10 °C with acceptable sensory scores [115].

These findings suggest that the fortification of dairy with lutein is viable. However, sensorial effects may present a challenge for marketing. Further studies should be performed in a variety of food matrices with different types of carotenoids and at varying concentrations to see if negative sensory effects can be prevented. Consideration of the type of dairy product is important, particularly with regard to color and taste. Sensory changes appear to be less noticeable in fortified yogurt than cheese. The stability of lutein is promising and suggests dairy is an ideal vehicle for the retention and delivery of carotenoids to the consumer. However, sensory-related challenges are apparent and must be overcome to ensure consumer interest.

### 5.3. Potential for Encapsulation in Dairy

The encapsulation of carotenoids in food as a method to optimize carotenoid delivery has recently been explored. Encapsulation presents the opportunity for a hydrophobic compound to be dispersed in a hydrophilic environment. Encapsulated delivery systems include biopolymeric and lipid-based nanocarriers (emulsions, nanoparticles, liposomes and hydrogels) in addition to microencapsulation techniques, spray and freeze drying [116,117]. This increases the type of food matrices available for functional food development, enabling foods with higher water content to be utilized [118]. Furthermore, encapsulation could enhance the stability, bioavailability and release of bioactive compounds [119]. Campo et al. examined the influence of carotenoid nanoencapsulation (nanoparticles and nanoemulsions) on the stability and sensory properties of yogurt [118]. The group demonstrated that zeaxanthin nanoparticles remained stable during storage. Zeaxanthin nanoparticles had greater retention, ~22%, than zeaxanthin nanoemulsions, ~16%. Furthermore, although bioavailability was higher in nanoemulsions, the authors suggest that the nanoparticles confer a greater advantage, attributing the low bioavailability of nanoparticles to a controlled release of zeaxanthin [118]. As noted by McClements et al. [119], a controlled or targeted release is a desirable characteristic of an encapsulated delivery system. Neither approach negatively impacted the sensory or physicochemical properties of the yogurt. Similarly, yogurt fortified with porcine gelatin encapsulated carotenoid extract from melons demonstrated β-carotene stability during storage [120]. Furthermore, the encapsulation of β-carotene in gelatin also promoted carotenoid solubility and contributed a positively perceived yellow color to the yogurt. The use of a water dispersible β-carotene and a spray-dried β-carotene powder in yogurt and pudding demonstrated the importance of the food matrix [121]. Micellarization was significantly higher for both forms in pudding (13.1% and 17%, respectively), than in yogurt (0.8% and 5.5%, respectively). However, encapsulation significantly reduced the incorporation into micelles regardless of the food matrix [121].

Encapsulated delivery of carotenoids could provide increased solubility and carotenoid stability with reduced adverse effects on the food sensory properties. However, the level of bioavailability remains low. This can in part be attributed to the choice of structuring material. Donhowe et al. noted that alginate and chitosan significantly reduced carotenoid micellarization in comparison with spray-dried β-carotene [121]. Similarly, Medeiros et al. reported superior solubility and stability following nanoencapsulation with porcine gelatin than whey protein isolate or whey protein concentrate [120]. The choice of structuring material and method of delivery should be considered when designing alternative carotenoid delivery systems with high bioavailability. Additionally, the interaction with the chosen food matrix must be evaluated. Consideration should be given to the loading capacity, safety and the commercial suitability of a proposed delivery system. Further exploration of the bioavailability of carotenoids in a dairy based food matrix following fortification with encapsulated carotenoids versus carotenoids in their free form should be carried out. Detailed reviews of alternative delivery systems of bioactive compounds and suggested systematic approaches to designing such delivery systems are available [119,122].

### 5.4. Agri-Food Waste—A Future Source for Dairy Fortification?

The by-products generated during the processing of fruit and vegetables are often discarded as waste despite being a rich source of bioactive compounds, including carotenoids [123]. The processing of fruit and vegetables in the United States and China results in approximately 15.0 and 32.0 million tons, respectively, of waste annually [124]. In recent years, the use of fruit and vegetable by-products, including the peel, pulp, pomace and seeds, has been identified as a novel source of carotenoids [125]. For instance, tomato peel contains lycopene, phytoene, phytofluene, β-carotene and lutein [126]. Furthermore, a concentration of 611.10 mg/100 g of lycopene has been reported from tomato pomace [127]. The major carotenoids reported in citrus fruit peel are α-carotene, β-carotene, lutein, zeaxanthin and β-cryptoxanthin at varying concentrations (11–204 mg/100 g) [128].

When considering food waste as a source of bioactive compounds, the choice of extraction technique is important. Oftentimes, particularly with lipophilic compounds such as carotenoids, toxic solvents are used as part of the extraction process [128]. Current studies are looking to shift to non-toxic solvents and even non-solvent-based techniques. Putnik et al. and Pattnaik et al. have recently reviewed the challenges facing extraction design and the current alternative methods under consideration [128,129].

There are limited studies to date on the fortification of dairy based foods with carotenoids from food waste by-products. However, the fortification of traditional Tunisian butter with lycopene from tomatoes waste (peel and seeds) has been demonstrated. The study found that fortification of butter with 400 mg of tomato waste by-product per kg reduced peroxide values during 60 days of storage at 4 °C. The fortified butter had improved shelf life and reduced lipid oxidation. Although, in the same study fortification at 800 mg/kg resulted in pro-oxidant activity [130]. A separate study by Rizk et al. noted that ice cream fortified with carotenoid from tomato peel waste improved anti-oxidant activity [126]. Furthermore, the ice cream fortified with 3% carotenoid extract had the highest scores during sensory evaluation (flavor, body and texture, melting and color) in comparison with both the control and other fortification concentration (1%, 2%, 4% and 5%). It is important to note that fortification at 4% and above resulted in a decline across all sensory scores [126]. As discussed in Section 5.3 the labile nature of carotenoids and the risk of degradation during processing has led to the encapsulation of carotenoids for improved stability and delivery. Šeregelj et al. has recently reported the encapsulation of carotenoids from carrot waste in alginate beads as part of a yogurt product at concentrations of 2.5 and 5 g per 100 g [131]. The yogurt was stored for 28 days at 4 °C and showed improved anti-oxidant activity whilst preserving the microbial and physiochemical properties of the yogurt.

The compositional value of fruit and vegetable waste by-products poses an opportunity for the farming, food and dairy industries to recycle waste products for functional food development with added nutritional value from a more sustainable source. Although limited, the findings to date suggest the potential for dairy fortification with carotenoids from food waste products due to their anti-oxidant properties. However, further research is warranted to establish the sensorial effects. The combination of agri-food waste with encapsulation techniques is a promising area for future consideration. As far as we are aware, no study has examined the bioaccessibility and bioavailability of such foods, this should be explored in future work.

### 5.5. The Effect of Processing on the Carotenoid Content and Bioaccessibility of Dairy Foods

Dairy products undergo various manufacturing and processing steps depending on the end product. The manufacturing process can alter the microstructure of food, influencing the retention of bioactive compounds (including carotenoids) and their bioaccessibility. The effect of processing on various dairy based food matrices is summarized in Table 2.

Hulshof et al. assessed the retention of β-carotene in Gouda and Edammer cheeses that were not artificially fortified. In Gouda, the β-carotene retention was 38% [62]. The β-carotene concentration of Gouda was significantly reduced during the first 12 days of the cheese-making process but was unaffected by ripening. In contrast, Edammer cheese had greater retention during the initial cheese making at 70%, than the ripening stage (40%) [62]. As previously mentioned (Section 5.2), the supplementation of Prato cheese with lutein survived the cheese-making process with high levels of retention (>90%) [112,115]. In Cheddar cheese, the lutein was added at the hooping stage to avoid potential degradation during processing but proved robust to the maturation stage [114]. Carotenoid retention during cheese making varies from 38 to 92.5%. Influencing factors include cheese and carotenoid type, supplementation or natural modification and the point of fortification. In semi-liquid dairy products, the naturally-derived carotenoid content had high levels of retention in yogurt at 92%, and similar high levels were observed for custard, buttermilk and cream [62]. Furthermore, 100% retention was observed in pasteurized full-fat milk. The fortification of yogurt with freeze-dried papaya and melon extract as carotenoid source was unaffected by fermentation but incurred a 20–27% reduction following pasteurization [132]. The retention of carotenoids in dairy products appears to be greater in semi-liquid dairy products and soft cheese as opposed to hard cheese. This is likely due to a combination of food matrix and processing conditions.

In bovine milk fortified with lutein via dietary supply, the effect of ultra-high-temperature (UHT) and high-temperature short-time (HTST) processing on lutein content was investigated. Findings by Wang et al. showed little effect of HTST on the lutein content of milk, whereas UHT resulted in reductions of 8%. Reductions were somewhat mitigated when lutein was co-supplemented with vitamin E. However, losses of 5% were still observed [102]. Thermal-based treatments are widely used for dairy processing as they are effective against microorganisms. These treatments alter the nutritional and sensorial properties of food products, therefore non-thermal alternatives are being considered. High-pressure processing (HPP) and high-intensity pulsed electric fields (HIPEF) of a functional beverage showed promising results, with greater carotenoid bioaccessibility than thermal treatment [110]. Thermal treatment resulted in reductions in bioaccessibility of up to 63%, regardless of the food matrix (milk, soy or water). In contrast, HIPEF of a milk-based functional beverage increased the carotenoid bioaccessibility by 15%. However, in a water-based matrix, all three treatments resulted in reductions, highlighting the influence of the food matrix. 

During development, functional foods will undergo various manufacturing steps and processing techniques. The impact of processing on the carotenoid content of food varies. When designing a fortified food, the stability of carotenoids within the food matrix and the subsequent bioavailability should be examined. The point of fortification and approach (natural modification, supplementation at farm level or during processing) and their interaction with processing should be investigated.

## 6. Carotenoids in Human Milk and Infant Formula

### 6.1. Carotenoid Content of Human Milk

In contrast to bovine milk, a greater variety of carotenoids that are more evenly distributed exist in human milk. In human milk, the major carotenoids are lutein, zeaxanthin, β-carotene, lycopene, α-carotene and β-cryptoxanthin [133,134,135]. Carotenoids are stored in the fat fraction. A number of influencing factors including inter- and intra-subject variation, milk type and diurnal variations have been reported [134]. Colostrum has 5 times the concentration of carotenoids as mature milk [136]. Hindmilk carotenoid content is 25% richer than mid- or foremilk [133]. Although not significant, a trend for higher levels in the morning than evening has been observed [133,134]. A multi-geographical study demonstrated that carotenoid profile is unique to country [137]. The variations observed between subjects, country and milk type are strongly associated with fat content and dietary effects. Factors that have no effect include BMI, number of years post-menarche and stage of lactation [135]. Similar to adults, carotenoid consumption has been linked with a number of health benefits in infants. In total, 50% of the carotenoid content in milk has provitamin A activity (α-carotene, β-carotene and β-cryptoxanthin) [137]. Lutein and zeaxanthin have been implicated in ocular development [138]. A correlation between infant serum zeaxanthin and infant macular pigment optical density has been demonstrated [139]. More recently, lutein has been identified as the predominant (59%) carotenoid in the infant brain. Furthermore, lutein levels were much lower in preterm infants, suggesting accumulation of lutein in the brain during the later stages of maturation in the womb [140]. These findings underline the importance of carotenoid consumption in infants. In the early stages of life, carotenoid ingestion is fully reliant on mothers’ own milk or formula alternatives. The influential nature of carotenoids on infant health stresses the importance of adequate dietary supply of carotenoids in infant formula.

### 6.2. Carotenoid Content of Infant Formula

When formula feeding is required, it is important that the nutritional composition of the formula mimics that of human milk. The carotenoid concentrations in non-fortified infant formulas vary, but are mainly low and not representative of human milk. Sommerburg et al. reported that the carotenoid profiles of infant formulas were much lower than that of human milk [136]. Only four out of eight formula preparations examined had any carotenoid content, with β-carotene in four and β-cryptoxanthin in three [136]. A higher concentration of carotenoids was recorded in infant formulas in Northern Ireland [141]. Lutein and zeaxanthin levels were present in 5 out of 6 formulas, although their levels varied greatly from 0.7 to 9.7 and 0.1 to 1.2 nmol/g fat, respectively. The authors attributed the high levels of lutein and zeaxanthin observed in some of the formulas to the fat source used, e.g., egg yolk, which has high xanthophyll content [141]. The low levels of carotenoids in formulas are reflected in infant plasma. Formula-fed infants had a median carotenoid plasma concentration of 14 µg/L in comparison with higher levels at birth of 24 µg/L or in the plasma of breastfed infants, at 32 µg/L [136]. It is apparent that carotenoid inclusion during the design of infant formula is not always considered. This can result in lower intake and accumulation of carotenoids in formula-fed infants. Due to the known bioactivity of carotenoids and their potential role in infant development, fortification of infant formula should be considered in future.

### 6.3. Fortified Infant Formula

In this section, we will discuss the development of fortified formula in terms of the bioaccessibility, bioavailability, safety and potential effects on infant development. It is important when designing infant formula that it is representative of the nutritional composition of breast milk. The primary carotenoids of interest for optimized infant nutrition are those with provitamin A activity or the xanthophylls, lutein and zeaxanthin, due to their implication in ocular and neural development.

#### 6.3.1. Bioaccessibility and Bioavailability of Fortified Infant Formula

Both human milk and lutein-fortified infant formula have similar bioaccessibility, 29% and 36%, respectively. However, the bioavailability of human milk was 4 times greater than the lutein-fortified formula [142]. These findings are supported by the higher plasma levels of carotenoids observed in breastfed infants in comparison with infants from a formula-fed group [15,143]. Bettler et al. reported that after 12 weeks, breastfed infants had mean lutein plasma levels of 69.3 µg/L in comparison with 11.3 µg/ L in the unfortified formula-fed group [15]. Furthermore, lutein-fortified formula resulted in an increase in the lutein serum levels of infants in a dose-dependent manner. Similarly, Mackey and colleagues reported higher carotenoid plasma concentrations in breastfed and fortified formula-fed infants, in comparison with the unfortified formula group [143]. Bettler et al. suggest that fortified infant formula needs 4 times the levels of lutein observed in human milk to have similar circulating levels in infant plasma [15]. Unfortified formula in both studies had low lutein levels of 13.7–20 µg/L. The successful fortification of infant formula with carotenoids has been demonstrated. However, infant formula has lower bioavailability than human milk. This should be taken into consideration when designing a formula to reflect the nutritive value of breast milk. The exact mechanisms responsible for higher bioavailability of human milk, despite similar bioaccessibility remain unclear. Perhaps, specific nutritional components or factors unique to human milk facilitate the optimized absorption and transportation of carotenoids. Greater understanding of the properties of human milk that allow for superior carotenoid delivery is needed to optimize a fortified formula product.

#### 6.3.2. Safety and Efficacy

When designing a fortified product for infant nutrition and health, the product must undergo the appropriate safety evaluations. Growth studies are key to evaluating the clinical suitability of infant formula and should monitor weight, length, GI tolerance and food intake [144].

The safety of lutein-fortified formula at concentrations of 200 µg/L has previously been reported [145]. Safety was evaluated through monitoring the occurrence of study events and blood chemistry analysis; all results fell within the normal range. The anthropometric results indicate similar growth effects in fortified and unfortified formulas, with regard to length, weight and head circumference. Overall, the lutein-fortified formula was well tolerated. Similarly, two fortified infant formulas with different concentrations of β-carotene, lycopene and lutein also proved safe and adequately supported growth in infants [143]. The total carotenoid concentration of the fortified formulas were 129.5 and 225.8 µg/L in comparison with much lower levels in the unfortified control, 27.8 µg/L. This study supports the safety and efficacy of a multi-carotenoid formula. An experimental formula fortified with lutein (11.4 µg/100 mL) supported the normal growth of infants [144]. Although the overall outcome was determined safe, three adverse side effects (rash; colic and abdominal pain; constipation) were observed in the experimental formula-fed group. However, the safety of lutein fortification at higher levels in other studies points to a different contributing factor of the experimental composition of the formula [144]. A study by Rubin et al. fortified an infant formula with β-carotene, lycopene and lutein. Again, the fortified formula was safe and effective for infant development [14]. Furthermore, lower plasma C-reactive protein (CRP) levels and greater rod photoreceptor sensitivity were observed in the fortified infant group in comparison with the control. These findings highlight a potential link between carotenoid intake and decreased inflammation. CRP is a commonly used marker of inflammation. Reductions in CRP following carotenoid supplementation suggest anti-oxidant and immunomodulatory effects of carotenoids. Additionally, findings implicate lutein in enhanced visual development of infants as supplemented formula improved rod photoreceptor sensitivity [14]. Clearly, the fortification of infant formula with carotenoids can produce a formula preparation that is safe, tolerated, and effective. Further clinical evaluations are required to confirm the potential health benefits of carotenoids during infant development.

## 7. Future Considerations

Overall, there is potential for a fortified milk product due to the associated health effects of carotenoids and the agreeable nature of the dairy food matrix. The carotenoid content of milk and milk products can be enhanced in three key ways, (1) naturally via farm management; (2) through supplementation of bovine feed; (3) by supplementation at the processing stage. The majority of studies to date have focused on fortification of dairy at the point of production. This strategy has the advantage of being able to incorporate the chosen carotenoid in a controlled and quantified manner. This mechanism may be further optimized by the inclusion of encapsulation techniques or recovering carotenoids from agri-food waste. The fortification of dairy at the point of farming has the advantage of being widely accessible to the farming industry, viewed more naturally by the consumer and is economically viable. However, there are limited studies to date on this mode of fortification and further evaluation in terms of bioaccessibility, bioavailability and stability following processing must be determined. As outlined by the findings presented in this review, the key factors that should be considered when developing a carotenoid-fortified dairy product include the method and point of milk fortification, the manufacturing and processing conditions required and perhaps most importantly, the composition and choice of food matrix. Additionally, the selection of carotenoid is not only important in terms of health associations but also due to differing polarity and interactions within the food structure. The bioaccessibility and bioavailability are primary concerns with promising but varying findings to date. Further scientific studies should establish the adequate dosage, the optimal delivery system and verify the stability and safety of carotenoid-fortified dairy products. Additionally, the effects of fortification on the nutritional value, sensory properties and bioactivity vary. Evidently, these properties and interactions must be assessed in a product-specific manner when designing a functional food suitable for commercial production.

Furthermore, carotenoids are of value in human milk with potential health benefits for the infant. Human milk and infant formula are the sole source of carotenoids for the developing infant during the first few months of life. The low levels of carotenoids associated with the available infant formulas highlights the need for fortification. Carotenoid fortification of infant formula provides a more representative source of nutrition for the infant when mother’s milk is unavailable. To date, findings show fortification is a safe and viable option that supports the normal growth of the infant. Further examination of fortification at high levels to achieve a similar serum level to breastfed infants should be performed.

This review highlights the growing body of evidence that the carotenoid content and profile of bovine milk can be modified for a product with increased nutritional and marketable value. Additionally, the potential to develop an infant formula that is more representative of human milk whilst preserving the safety and efficacy of the formula is apparent. However, further research is required to establish the clinical benefits associated with a fortified formula or a dairy functional food. 

## Figures and Tables

**Table 1:** The lipid transporters implicated in the intestinal absorption of carotenoids.

Carotenoid	Lipid Transporter	References
β-Carotene	SR-BI, NPC1L1	[43,44,45]
α-Carotene	SR-BI, NPC1L1	[43,44]
β-Cryptoxanthin	SR-BI, NPC1L1	[43,44]
Lutein	SR-BI, NPC1L1, CD-36	[44,46,47,48]
Zeaxanthin	SR-BI, NPC1L1	[44,45]
Phytoene	SR-BI	[49]
Phytofluene	SR-BI	[49]
Lycopene	SR-BI, NPC1L1 *, CD-36	[44,47,50]

Asterisk (*) indicates that at least one study has not implicated this lipid transporter.

**Table 2:** The effect of processing on the carotenoid content of dairy products.

Food Matrix	Carotenoid	Processing Technique	Carotenoid Retention (%)	Reference
Gouda cheese	β-Carotene	Cheese making (initial 12 days)	↓ 62%	[62]
		Ripening (26 weeks)	↓ 9%	
Edammer cheese	β-Carotene	Cheese making (initial 12 days)	↓ 30%	[62]
		Ripening (20 weeks)	↓ 60%	
Prato cheese	Lutein	Prato cheese making (after milk pasteurization and at milk coagulation)	↓ <10%	[112,115]
Cheddar cheese	Lutein	Ripening (24 weeks)	x	[114]
Yogurt	β-Carotene, β-Cryptoxanthin, Lycopene	Pasteurization	↓ 20–27%	[132]
		Fermentation	↓ <3%	
Milk	Lutein	HTST	↓ <3%	[102]
		UHT	↓ 8%	
Milk	Lutein + Vitamin E	HTST	↓ <3%	[102]
		UHT	↓ 3%	
Milk	β-Carotene	Pasteurization	x	[62]

(↓) indicates reduction. (x) indicates no change, 100% retention. HTST = High-temperature short-time. UHT = Ultra-high-temperature.
